# Bisphenol A Exposure Disrupts Genomic Imprinting in the Mouse

**DOI:** 10.1371/journal.pgen.1003401

**Published:** 2013-04-04

**Authors:** Martha Susiarjo, Isaac Sasson, Clementina Mesaros, Marisa S. Bartolomei

**Affiliations:** 1 Department of Cell and Developmental Biology, University of Pennsylvania Perelman School of Medicine, Philadelphia, Pennsylvania, United States of America; 2 Center of Excellence in Environmental Toxicology, University of Pennsylvania Perelman School of Medicine, Philadelphia, Pennsylvania, United States of America; 3 Department of Obstetrics and Gynecology, University of Pennsylvania Perelman School of Medicine, Philadelphia, Pennsylvania, United States of America; 4 Centers for Cancer Pharmacology and Excellence in Environmental Toxicology, Department of Pharmacology, University of Pennsylvania Perelman School of Medicine, Philadelphia, Pennsylvania, United States of America; The Babraham Institute, United Kingdom

## Abstract

Exposure to endocrine disruptors is associated with developmental defects. One compound of concern, to which humans are widely exposed, is bisphenol A (BPA). In model organisms, BPA exposure is linked to metabolic disorders, infertility, cancer, and behavior anomalies. Recently, BPA exposure has been linked to DNA methylation changes, indicating that epigenetic mechanisms may be relevant. We investigated effects of exposure on genomic imprinting in the mouse as imprinted genes are regulated by differential DNA methylation and aberrant imprinting disrupts fetal, placental, and postnatal development. Through allele-specific and quantitative real-time PCR analysis, we demonstrated that maternal BPA exposure during late stages of oocyte development and early stages of embryonic development significantly disrupted imprinted gene expression in embryonic day (E) 9.5 and 12.5 embryos and placentas. The affected genes included *Snrpn, Ube3a, Igf2, Kcnq1ot1, Cdkn1c*, and *Ascl2*; mutations and aberrant regulation of these genes are associated with imprinting disorders in humans. Furthermore, the majority of affected genes were expressed abnormally in the placenta. DNA methylation studies showed that BPA exposure significantly altered the methylation levels of differentially methylated regions (DMRs) including the *Snrpn* imprinting control region (ICR) and *Igf2* DMR1. Moreover, exposure significantly reduced genome-wide methylation levels in the placenta, but not the embryo. Histological and immunohistochemical examinations revealed that these epigenetic defects were associated with abnormal placental development. In contrast to this early exposure paradigm, exposure outside of the epigenetic reprogramming window did not cause significant imprinting perturbations. Our data suggest that early exposure to common environmental compounds has the potential to disrupt fetal and postnatal health through epigenetic changes in the embryo and abnormal development of the placenta.

## Author Summary

BPA is a widely used compound to which humans are exposed, and recent studies have demonstrated the association between exposure and adverse developmental outcomes in both animal models and humans. Unfortunately, exact mechanisms of BPA–induced health abnormalities are unclear, and elucidation of these relevant biological pathways is critical for understanding the public health implication of exposure. Recently, increasing data have demonstrated the ability of BPA to induce changes in DNA methylation, suggesting that epigenetic mechanisms are relevant. In this work, we study effects of BPA exposure on expression and regulation of imprinted genes in the mouse. Imprinted genes are regulated by differential DNA methylation, and they play critical roles during fetal, placental, and postnatal development. We have found that fetal exposure to BPA at physiologically relevant doses alters expression and methylation status of imprinted genes in the mouse embryo and placenta, with the latter tissue exhibiting the more significant changes. Additionally, abnormal imprinting is associated with defective placental development. Our data demonstrate that BPA exposure may perturb fetal and postnatal health through epigenetic changes in the embryo as well as through alterations in placental development.

## Introduction

Perturbed gestation affects fetal growth and development, resulting in a predisposition to diseases [1]. The “developmental origin of adult disease” hypothesis was originally formulated based on clinical data linking low birth weight to increased risks for adult onset cardiovascular and metabolic disorders. The hypothesis has been supported by a growing number of human diseases associated with unfortunate events during pregnancy including drug exposure [2], chemical exposure [3], prenatal stress [4], and maternal caloric restriction [5]. The observed phenotypes in the fetus were frequently accompanied by altered gene expression [2]–[5]. Although the available data strongly suggest that the environment significantly impacts fetal development, exact molecular mechanism(s) linking diseases and early life events remain unclear despite significant research.

Accumulating evidence implicates the role of epigenetics in mediating gene-environment interactions. Studies have demonstrated the ability of environmental factors including food constituents [6], prenatal famine [7], and endocrine disruptors [8]–[10] to alter global or gene-specific DNA methylation patterns. DNA methylation, the addition of a methyl group to the cystosine residue of DNA, is an epigenetic mechanism that regulates genes important for a wide range of biological processes. DNA methylation at regulatory regions typically leads to gene repression and altered patterns are associated with developmental defects, tumors and cancer [11], [12]. Furthermore, early mammalian development represents a vulnerable window as the genome undergoes dynamic changes of DNA methylation [13] and environmental factors that alter these epigenetic reprogramming events may adversely impact growth and development.

Several environmental factors that can perturb epigenetic reprogramming events in the embryo have been reported [14]–[17]. These factors include conditions or techniques related to embryo culture used in assisted reproductive technologies (ART). Although the number of babies that are conceived through ART is growing, these individuals represent a relatively small percentage (1–4%) of babies in industrialized countries [18]. In the current study, we investigated a widely used environmental compound that has the ability to alter DNA methylation [9]. The compound, bisphenol A (BPA), is essentially ubiquitous in the environment and can be found in various consumer products including polycarbonate plastics, resin and paper receipts [19]. Most humans are exposed to BPA [19] with estimated blood levels ranging from 0.5 to 10 ng/mL [20]. Importantly, BPA is a significant public health issue as early developmental exposure is linked to abnormal brain function, metabolism, reproduction, behavior, and immune system in model organisms [19]. Aside from its estrogenic properties and ability to bind to estrogen receptors [21], molecular mechanisms related to BPA action are not well elucidated and most likely, are complex based on the broad range of phenotypes related to exposure. Recently, it has been reported that BPA exposure can alter the mouse and rat epigenome [9], [22]–[24]. Jirtle and coworkers [9] reported that BPA exposed mice exhibited decreased DNA methylation at two mouse metastable loci while other studies [22]–[24] found DNA methylation changes at endogenous loci relevant to prostate, brain and uterine development. These studies [22], [24] investigated BPA effects when exposure occurred during mid-gestation or neonatal period. As we hypothesized that the earlier period of post-fertilization development is the most sensitive window due to its inherent epigenetic reprogramming, it would be important to determine potential impacts of BPA-induced epigenetic perturbations on developmentally relevant genes in the conceptus when the exposure occurs during this window.

In this study we ask whether BPA exposure in mice can alter imprinted gene expression and DNA methylation. Genomic imprinting in mammals results in the epigenetic modification of a small number of developmental genes such that a single parental allele is preferentially or exclusively expressed [25]. The use of genomic imprinting to study the developmental effects of BPA on the epigenome is highly suitable as differential DNA methylation is a well-characterized imprinting mechanism [25]. Furthermore, imprinted genes are developmentally critical as they are essential for embryonic, placental and postnatal growth and aberrant imprinting causes cancer and human disorders including Beckwith-Wiedemann Syndrome (BWS) and Angelman Syndrome (AS) [26]. Here we perform extensive characterization of the allelic and total expression of imprinted genes on mouse chromosome 7 and on DNA methylation of the associated imprinting control regions (ICRs) as well as genome-wide methylation in mice exposed to two physiological doses of BPA during early embryonic development. Our data show that BPA disrupts proper expression of imprinted genes in a gene- and tissue-specific manner when exposure occurs early in development. Specifically, we observe significant perturbations at the *Snrpn, Ube3a, Igf2*, *Kcnq1ot1* and *Cdkn1c* loci but not at the *H19* and *Peg3* genes. Moreover these effects are DNA methylation-dependent as demonstrated by analysis at the *Snrpn* ICR and *Igf2* differentially methylated region (DMR) 1, although our data also suggest that other epigenetic mechanisms cannot be excluded. Further studies reveal that aberrant imprinting in BPA exposed mice is associated with abnormal placental development. In contrast, BPA treatment of pregnant females outside of the epigenetic reprogramming window, i.e., from embryonic day (E) 5.5 to 12.5, had no observable effect on genomic imprinting. These findings indicate that exposure to the common environmental compound at low, physiologically relevant doses during the earliest stage of embryonic development may disrupt epigenetic reprogramming events and perturb developmental gene expression.

## Results

### BPA exposure leads to aberrant expression of imprinted genes on mouse chromosome 7

Dolinoy et al. reported that BPA exposure in pregnant viable yellow agouti (*A^vy^*) mice resulted in offspring with reduced DNA methylation at 9 CpGs within the intracisternal A particle (IAP) retrotransposon locus and altered *Agouti* expression [9]. More recent studies reported DNA methylation changes at various loci in BPA exposed rats and mice [22]–[24]. Because expression of imprinted genes is typically regulated by allele-specific methylation at ICRs, we hypothesized that BPA exposure would perturb expression of imprinted genes.

**Figure 1:**
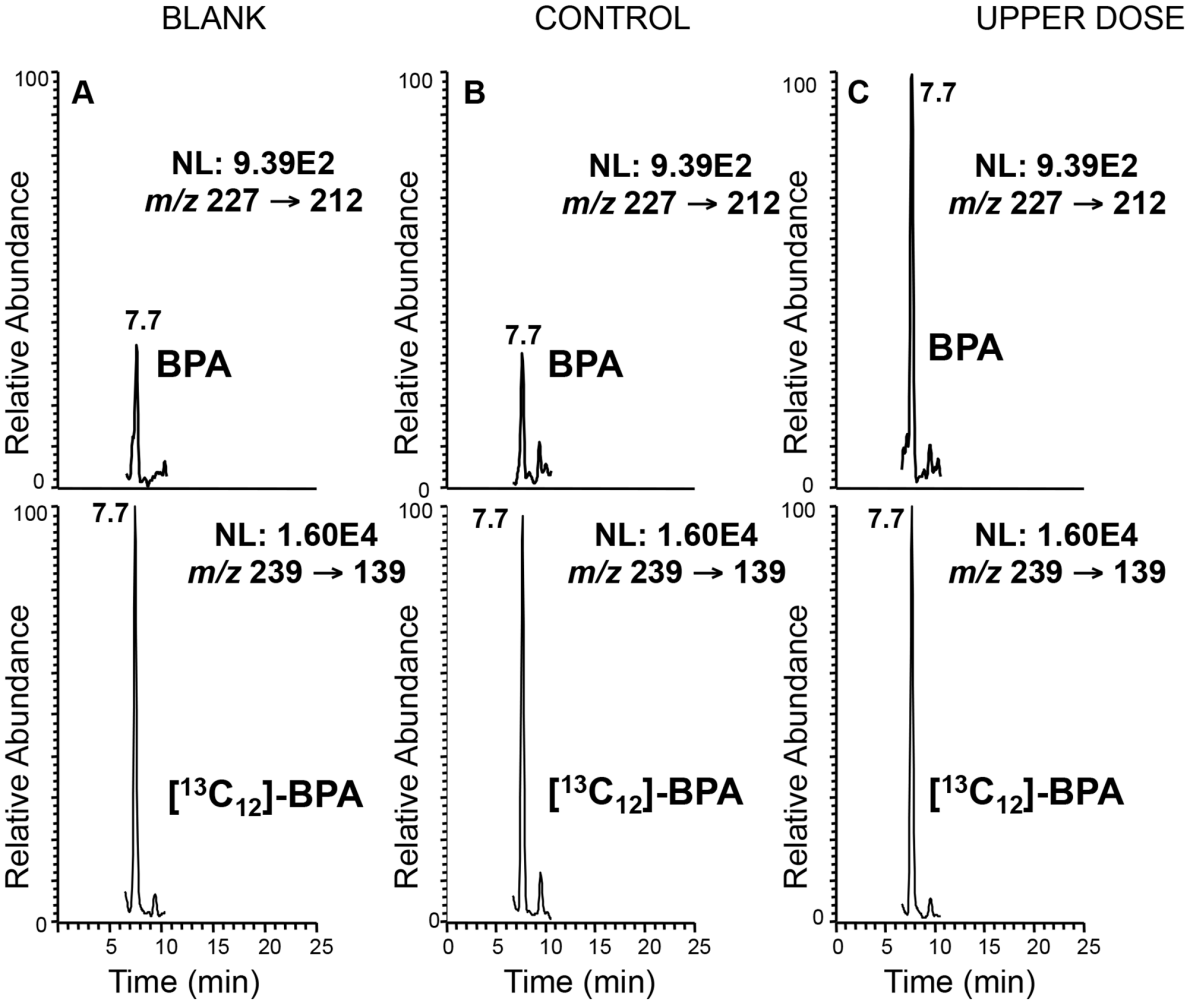
Representative LC-SRM/MS chromatograms demonstrating significantly higher BPA level in serum from treated mice. Top panels represent the chromatograms for unlabeled BPA and lower panels labeled BPA ([^13^C_12_]-BPA) spiked prior to sample extraction to quantify BPA levels in serum from representative control (B) and upper dose treated mice (C). Blank sample used as control for storage and extraction is indicated in (A). Y-axis represents relative abundance of signal intensity and X-axis retention time in minute. Differences in BPA concentrations were determined based on peak heights (see Materials and Methods). NL = normalized levels of intensity; *m/z = *mass to charge ratio.

**Figure 2:**
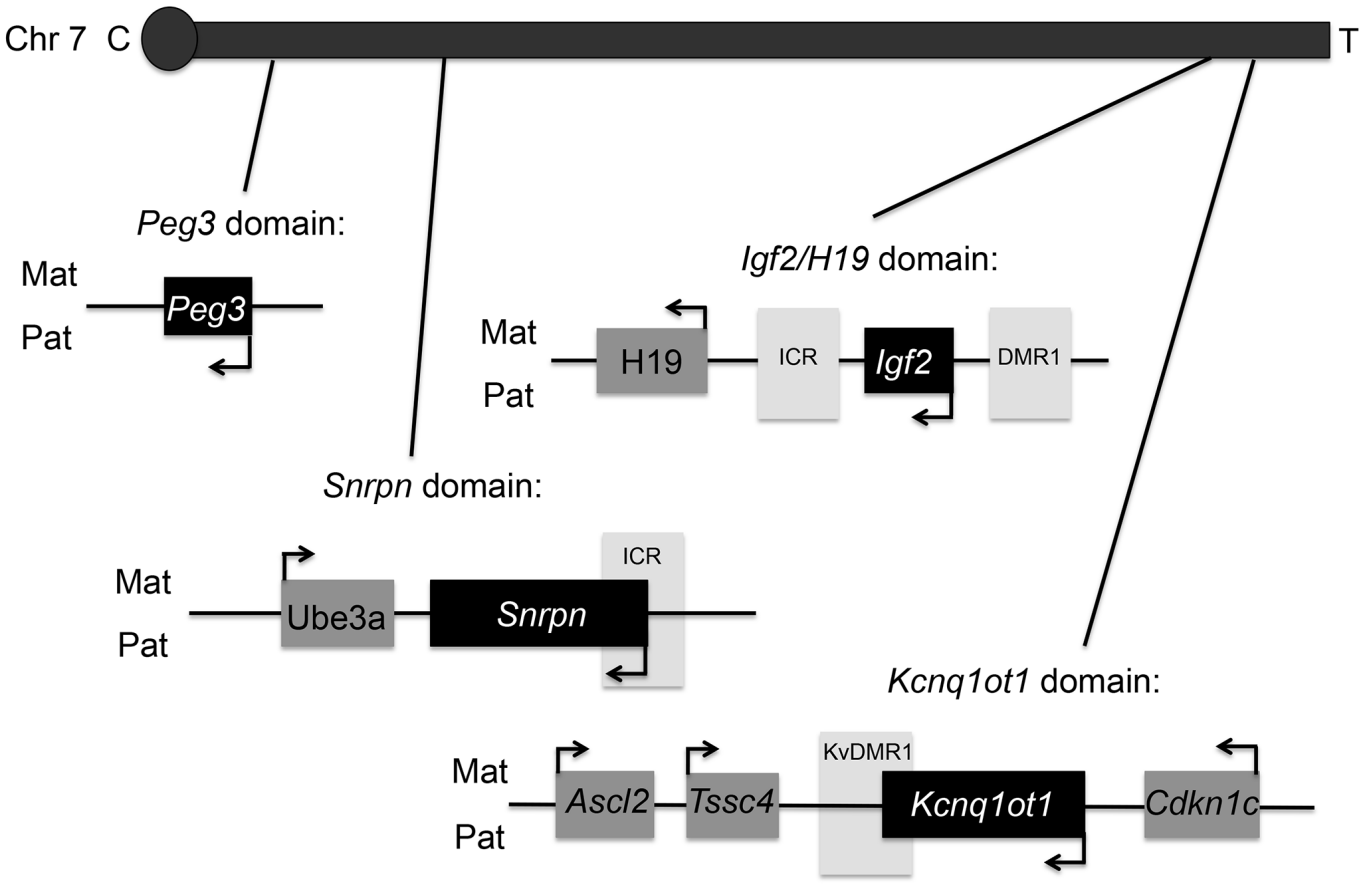
Map of mouse chromosome 7 showing imprinted domains analyzed in the current work is illustrated. Black = paternally expressed genes; dark grey = maternally expressed genes; and light grey = differentially methylated regions (DMRs). Arrows indicated direction of gene transcription. C = centromere and T = telomere. Not drawn to scale.

To test the hypothesis, we analyzed F1 hybrid mice generated from reciprocal matings of the C57BL/6 (B6) and the B6 (CAST7) or C7 mice. We treated female mice starting from 2 weeks prior to mating until E9.5 with three different doses of dietary BPA: 0 (control), 10 mg/kg bw/day (upper dose) and 10 µg/kg bw/day (lower dose). The upper dose treatment paradigm was similar to the method employed by Jirtle and coworkers, except that we analyzed the offspring before birth [9]. The lower dose represented safe human exposure level, i.e., at or below 50 µg/kg bw/day [20]. BPA serum measurement revealed that the treatment paradigm resulted in significantly increased level of unconjugated BPA in pregnant mice from the upper dose group compared to controls (2.0±0.4 ng/mL vs. 0.7±0.2 ng/mL, respectively; n = 3; P<0.01; Figure 1). Serum BPA detected in pregnant mice from the lower dose exposure group showed a trend towards increased levels but the difference was not statistically significant compared to controls (data not shown). Embryonic and placental tissues from the F1 hybrid offspring were subsequently analyzed at E9.5 for allele-specific transcription of imprinted genes located in multiple domains, including the *Peg3*, *Snrpn, H19/Igf2 and Kcnq1* domains, on mouse chromosome 7 (Figure 2). We initially analyzed E9.5 because many imprinted genes display parent-of-origin specific expression in the whole embryo at this time. Additionally, the embryo and placenta are sufficiently differentiated so that risk for cross contamination is low, and more importantly, maternal tissue contamination in decidua-removed placenta is minimal compared to later stages.

**Figure 3:**
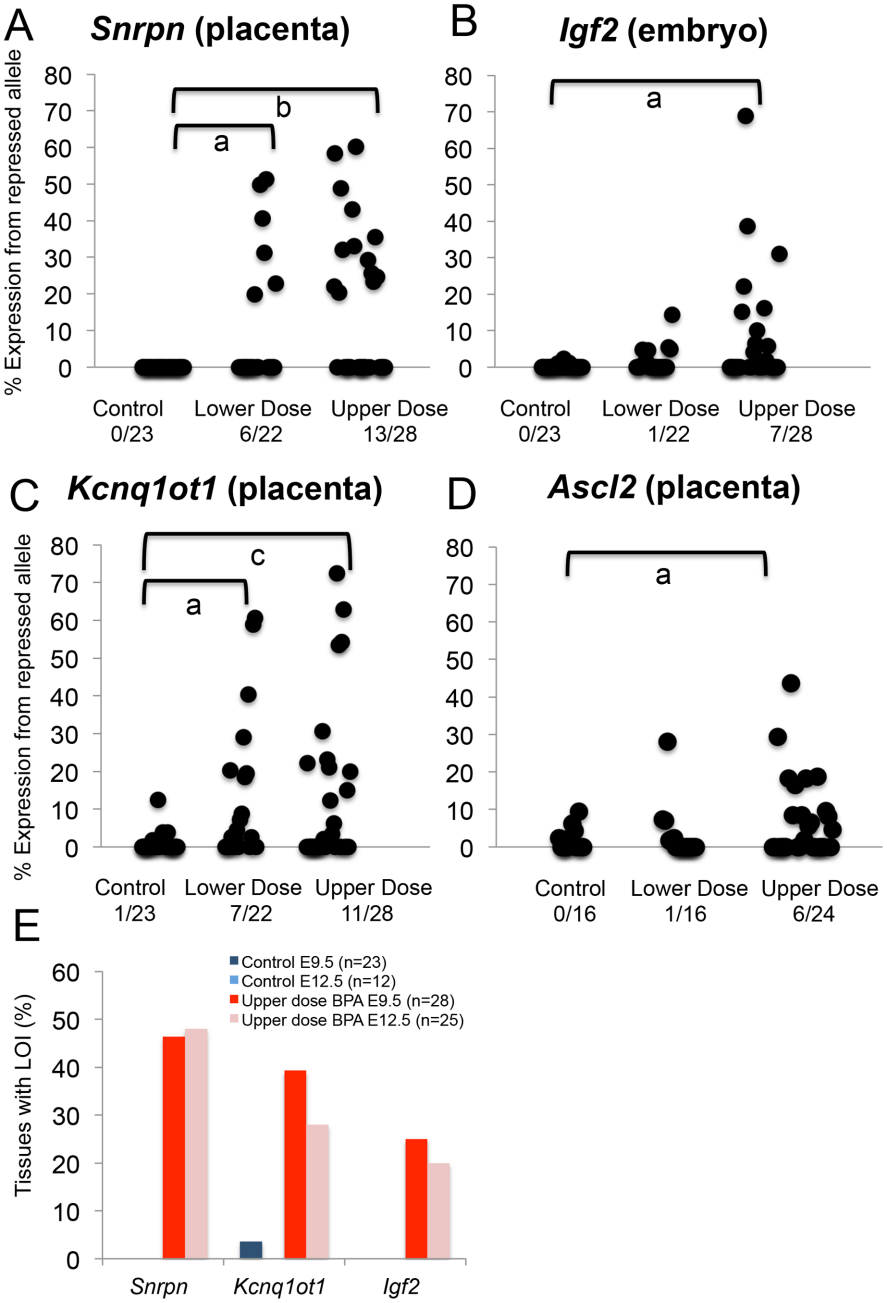
Allele-specific expression studies showed increased proportion of tissues with loss of imprinting following BPA exposure. (A–D) Each black circle represents an individual E9.5 embryo (*Igf2*) or placenta (*Snrpn*, *Kcnq1ot1* and *Ascl2*). Y-axis indicates percentage of total mRNA expression derived from the repressed allele. Loss of imprinting (LOI) or biallelic expression was called when the repressed allele exhibited ≥10% of total expression. Data from control (n = 23), lower dose (n = 22) and upper dose (n = 28) exposure groups are shown for (A) *Snrpn*, (B) *Igf2*, (C) *Kcnq1ot1* and (D) *Ascl2*; a = P<0.05; b = P<0.01 and c = P<0.001. Number of tissues (out of total examined) showing LOI is indicated below each exposure group. In (E), percentages of placentas (*Snrpn and Kcnq1ot1*) and embryos (*Igf2*) showing LOI at E9.5 and E12.5 are compared.

Upper dose BPA exposure disrupted the parental specific, monoallelic expression of the *Snrpn, Igf2 and Kcnq1ot1* genes in a tissue-specific manner (Figure 3A–3C). Upper dose BPA exposure resulted in significantly more placentas exhibiting biallelic expression of the paternally expressed *Snrpn* gene (Figure 3A); no significant effect was observed in the embryo (Table S1). Loss of imprinting (LOI) occurred in 13/28 placentas from upper dose BPA exposed mice compared to 0/23 in controls (P<0.001; Figure 3A). Analysis of the placentas exhibiting LOI in the upper dose BPA group showed that the normally repressed maternal *Snrpn* allele contributed 20.3–60.2% of total expression (Figure 3A). At the *Igf2* locus, upper dose BPA exposure significantly resulted in LOI in 7/28 embryos as compared to 0/23 in controls (Figure 3B; P<0.05), whereas no effects were observed in the placenta (Table S1). Among the upper dose BPA exposed embryos showing LOI, *Igf2* expression from the normally repressed maternal allele ranged from 10.0–68.9% (Figure 3B). Analysis of the placenta-specific P0 promoter (*Igf2* P0) revealed normal, monoallelic expression in placentas from both control and upper dose exposure groups (data not shown). Interestingly, although *Igf2* and *H19* were located in the same domain and are coordinately regulated through a shared ICR [27], the maternally expressed *H19* gene remained monoallelically expressed (Table S1). We also tested allelic expression of the paternally expressed *Kcnq1ot1* (located in the *Kcnq1* domain) and found 11/28 placentas showing LOI in the upper dose BPA group vs. 1/23 in the control (Figure 3C; P<0.05). No significant perturbation was detected in the embryo (Table S1). Expression of the normally repressed maternal allele in the placentas showing *Kcnq1ot1* LOI ranged from 12.3–72.3%; in the control group, one placenta with LOI had 12.4% of total expression from the normally repressed maternal allele (Figure 3C). At the *Peg3* locus, no significant effect was observed in conceptuses exposed to upper dose BPA (Table S1).

Lower dose BPA exposure resulted in mostly normal, monoallelic expression, except at the *Snrpn* and *Kcnq1ot1* loci in the placenta. At the *Snrpn* locus, 6/22 placentas exhibited LOI (compared to 0/23 in controls; P<0.05) with expression of the normally repressed maternal allele ranging from 19.8–51.3% (Figure 3A). At the *Kcnq1ot1* locus, 7/22 placentas in the lower dose group showed LOI (compared to 1/23 in the control group; P<0.05; Figure 3C) with expression of the repressed maternal allele ranging from 18.5–60.7%. *Igf2*, *H19* and *Peg3* (Figure 3B and Table S1) and *Igf2 P0* (data not shown) were normally expressed in the embryonic tissues and/or placentas from mice exposed to lower dose BPA.

To determine whether BPA exposure affected genomic imprinting in embryonic organs, we analyzed allele-specific expression of the *Snrpn, Igf2* and *Kcnq1ot1* genes in E12.5 conceptuses. We first analyzed LOI frequencies at these loci in upper dose BPA and control exposed E12.5 whole embryos and placentas. Similar frequencies of imprinting abnormalities were observed in E12.5 as compared to E9.5 in the placenta: 12/25 and 7/25 exhibited LOI at the *Snrpn* and *Kcnq1ot1* loci, respectively (P>0.05 as compared to 13/28 and 11/28 in E9.5 placentas, respectively; Figure 3E and Table S2) – no significant effect was detected at the *Igf2, H19 and Peg3* loci in the placenta (data not shown). In E12.5 whole embryos, 5/25 showed LOI at the *Igf2* locus (P>0.05 as compared to 7/28 at E9.5; Figure 3E and Table S2) but no LOI observed at the *Snrpn* and *Kcnq1ot1* genes (data not shown). Our analysis had therefore demonstrated similar LOI rates in E12.5 vs. E9.5 conceptuses.

Next, we analyzed LOI rates in E12.5 embryonic organs including the heart, liver, brain and kidney. No significant imprinting abnormalities were observed in these organs at the *Snrpn*, *Kcnq1ot1* and *Igf2* loci (n = 16–17 mice analyzed; data not shown), except in the brain at the *Snrpn* locus (4/16 in upper dose BPA vs. 0/17 in control; P = 0.05).

Imprinting analyses were conducted in maternal decidua-removed placentas. Nevertheless, a recent study indicated that these tissues contain significant levels of some RNAs from maternal cells that can bias analysis of maternally expressed imprinted genes [28]. As we observed abnormal imprinting of maternally expressed genes (e.g., *Snrpn and Kcnq1ot1*) in the placenta, we subsequently determined whether BPA exposed placentas had more maternal cell contamination as compared to controls by measuring total expression of the *Gatm, Tfpi2* and *Ampd3* genes; these genes were shown to be highly expressed in the maternal decidua as compared to placenta [28]. Total expression of the *Gatm, Tfpi2* and *Ampd3* genes relative to the reference genes *Arppo* and *Gapdh* in upper dose BPA exposed (n = 5) and control E12.5 placentas (n = 5) was not significantly different (P>0.05; data not shown) indicating that the increased LOI rates observed in BPA exposed placentas were not associated with increased maternal cell contamination.

In total, we analyzed 4–6 litters per exposure group from both C7XB6 and B6XC7 matings at E9.5 and 2–4 litters at E12.5 (see Table S1, S2 for complete list). We did not observe significant LOI differences between C7XB6 and B6XC7 matings for any of the tested genes (Table S1, S2). Although similar LOI rates were observed in E9.5 (Table S1) and E12.5 (Table S2) whole embryos and placentas, significant litter-specific effects were observed in the latter stage litters (P = 0.02 for *Snrpn*) but not in the earlier (P>0.05 for all genes tested). In E12.5 brains, however, we did not observe significant litter-specific effects for *Snrpn* LOI.

### BPA exposure outside of the epigenetic reprogramming window does not lead to imprinting defects

Our exposure paradigm coincided with the latter stage of oocyte development during DNA methylation acquisition [29] and the earliest stage of embryonic development during the extensive epigenetic reprogramming in the genome [13]. To determine whether exposure outside of this window leads to aberrant imprinting, we treated pregnant mice with control and upper dose BPA from E5.5 to E12.5 and determined the allele-specific expression of the *Snrpn*, *Igf2* and *Kcnq1ot1* genes in the whole embryo and placenta. Our data demonstrated no significantly different rates of LOI at these loci in both the embryonic and placental tissues of upper dose BPA exposed E12.5 mice as compared to the control group (n = 12 and 20 mice analyzed in 2 control litters and 4 upper dose BPA litters, respectively; data not shown).

### Aberrant allelic expression of imprinted genes is associated with significant changes in total expression

**Figure 4:**
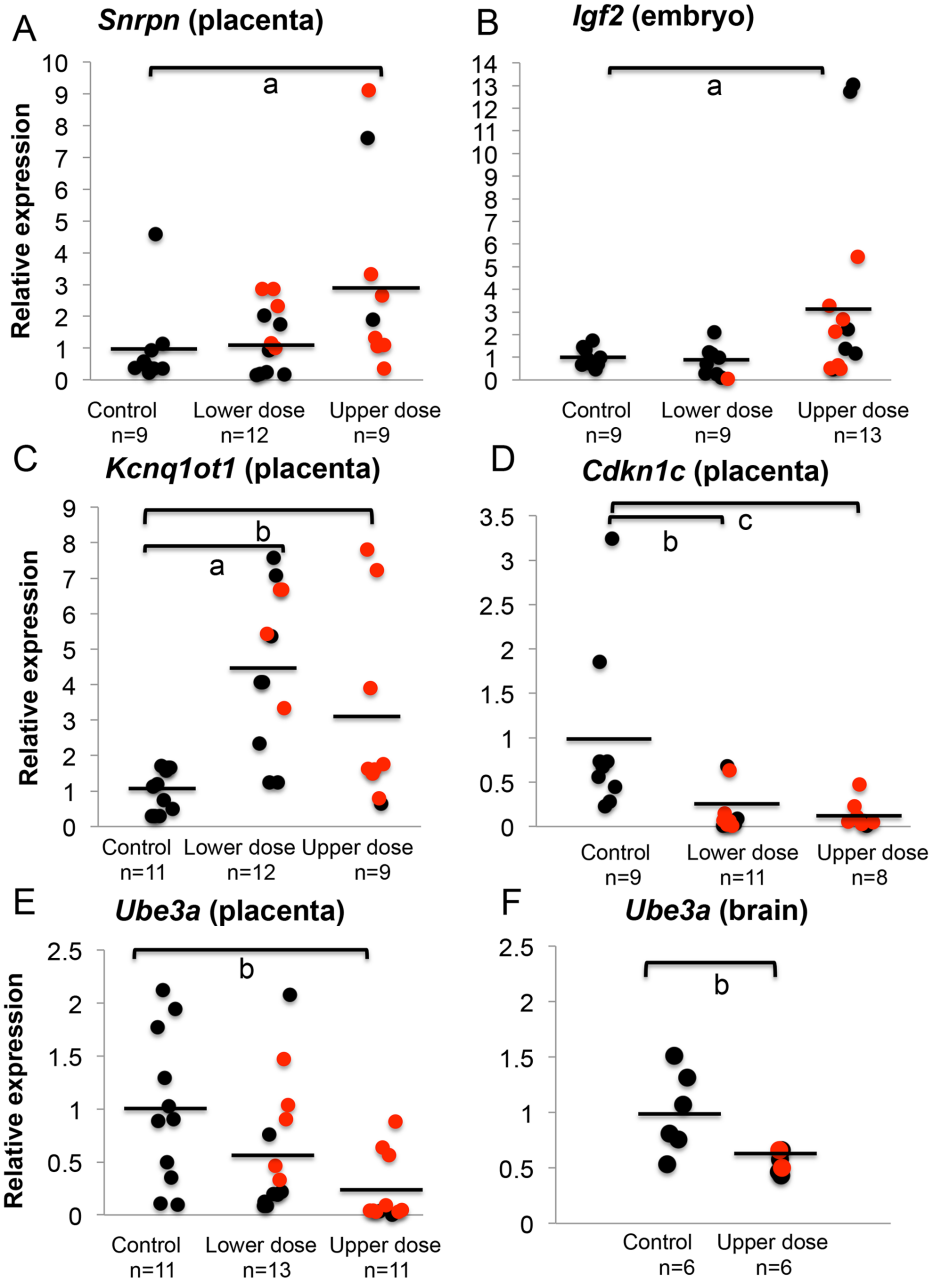
BPA exposure altered total mRNA expression of imprinted genes relative to reference genes. Samples from control, lower dose and upper dose exposure groups were analyzed for total expression of the imprinted (A) *Snrpn*, (B) *Igf2*, (C) *Kcnq1ot1*, (D) *Cdkn1c* and (E and F) *Ube3a* genes with sample sizes (n) indicated. Assayed samples included embryos (*Igf2*), placentas (*Snrpn, Kcnq1ot1, Cdkn1c*, and *Ube3a*) or brains (*Ube3a*) showing normal, monoallelic (black circle) or biallelic expression (red circle). Average total expression in each exposure group is indicated with a black horizontal line; a = P<0.05; b = P<0.01 and c = P<0.001.

To address whether total expression of imprinted genes was impacted by BPA exposure, we re-analyzed the embryonic and placental tissues from the offspring of pregnant mice treated with control and BPA diets starting from pre-mating until E9.5. We quantified expression of the *Snrpn, Igf2*, and *Kcnq1ot1* genes relative to the reference genes *Arppo* and *Gapdh*. Quantitative real time PCR analysis of *Snrpn* revealed that placentas from mice exposed to upper dose BPA had a significant increase of mean total RNA compared to control placentas (3.2±3.1 vs. 1.0±1.3, respectively; n = 9 in each group; P<0.05; Figure 4A). In the lower dose group, mean total expression was not significantly different when placentas with and without LOI were analyzed together (1.3±1.0; n = 12; P>0.05; Figure 4A); however, lower dose BPA exposed placentas exhibiting LOI had significantly higher *Snrpn* expression (2.0±0.8; n = 5) when compared to samples with normal, monoallelic expression (0.7±0.6; n = 7; P<0.01). At the *Igf2* locus, average total expression was also significantly increased in upper dose BPA exposed embryos (3.6±4.3; n = 13) compared to controls (1.0±0.4; n = 9; P<0.05; Figure 4B); no significant change was observed in embryos from the lower dose group (0.8±0.7; n = 9; P = 0.19; Figure 4B), consistent with their normal allelic expression (Figure 3B). Total *H19* expression in both lower and upper dose groups was not different from controls (data not shown). Furthermore, biallelic expression of *Kcnq1ot1* in the placentas from the upper dose group was associated with significant increased total expression as compared to controls (3.0±2.7 vs. 1.0±0.5, respectively; n = 9 and 11; P<0.01; Figure 4C); similar observation was found in the lower dose group (4.5±2.2 vs. 1.0±0.5 in controls; n = 12 and 11, respectively; P<0.05; Figure 4C).

One common feature of imprinted genes is that they are typically located in clusters throughout the genome [30]. The clusters contain maternally and paternally expressed genes, a non-coding RNA and an ICR that is subject to differential methylation, hence often called the DMR [30]. Because imprinted genes located in the same domain were often co-regulated [30], we further examined whether BPA also impacted expression of other genes located in the affected domains by analyzing their allelic and/or total expression. At the *Kcnq1* domain, we measured total expression of *Cdkn1c*, a cell cycle inhibitor gene. Placentas from mice exposed to upper dose BPA showed significant reduction in total *Cdkn1c* expression compared to control placentas (0.1±0.2 vs. 1.0±0.9, respectively; n = 8 and 9; P<0.05; Figure 4D). The lower dose group (n = 11) was similarly impacted (0.2±0.2 vs. 1.0±0.9 in controls; P<0.01; Figure 4D). These observations suggested that effects of BPA were not limited to the *Kcnq1ot1* gene in this domain. Analysis of the allelic expression of *Cdkn1c* in exposed tissues revealed normal monoallelic expression (data not shown).

At the *Snrpn* domain, we tested expression of the maternally expressed *Ube3a* gene (∼1110 kb downstream; Figure 2). Interestingly, total expression of *Ube3a* in upper dose BPA exposed placentas (0.2±0.3; n = 11) was significantly reduced as compared to controls (1.0±0.7; n = 11; P<0.01; Figure 4E). The difference observed in the lower dose group, however, was not statistically significant compared to controls (0.6±0.6; n = 13; P = 0.08). It is unclear why *Ube3a* was downregulated in the placenta as it is a brain-specific imprinted gene and was transcribed biallelically in both control and BPA exposed placentas (data not shown). We subsequently tested total expression of *Ube3a* gene in E12.5 brain from control and upper dose BPA exposed mice and found that brain tissues from upper dose BPA exposure group expressed significantly lower levels of *Ube3a* (0.6±0.1; n = 6) than controls (1.0±0.4; n = 6; P<0.01; Figure 4F).

To account for the potentially non-normal distribution of our total expression measurements (Figure 4A–4F), all data have been alternatively analyzed using a non-parametric statistical test (Materials and Methods). We found that BPA exposure-induced changes in total expression of the imprinted genes were statistically significant using this method (Figure S1A–S1F).

### BPA exposure alters average DNA methylation levels at the differentially methylated regions of imprinted loci

**Figure 5:**
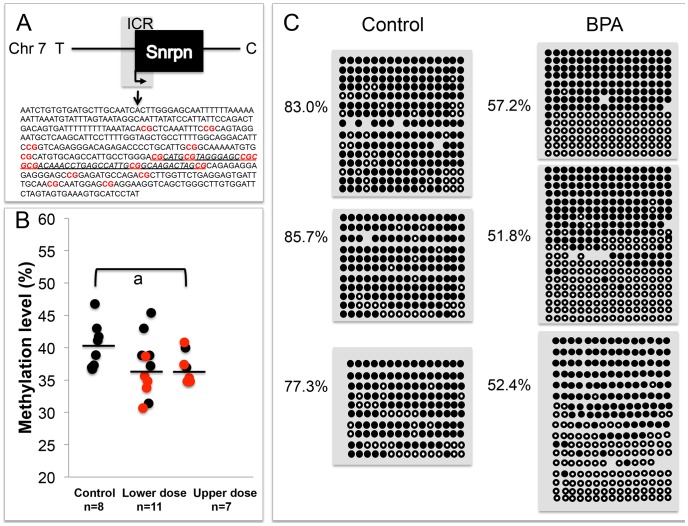
Analysis in control and upper dose BPA exposed placentas indicated that exposure significantly reduced methylation. The *Snrpn* ICR is depicted in (A), including the sequences analyzed in the (B) pyrosequencing and (C) bisulfite sequencing assays. A. 16 CpG sites (highlighted in red and bold) located in a 451 bp region of the *Snrpn* ICR were assayed by bisulfite sequencing. The underlined genomic sequence containing 7 CpGs represents the region analyzed by pyrosequencing. B. Pyrosequencing data in control, lower dose and upper dose BPA exposure are shown with samples exhibiting monoallelic (black circles) or biallelic expression of *Snrpn* (red circles). Y-axis represents percentage of total methylation. Black horizontal line in each exposure group indicates average methylation. Sample sizes analyzed in each exposure group are shown; a = P<0.05 when analyzed through ANOVA but not significant through Kruskal-Wallis. C. Three placentas from both control and upper dose BPA exposure groups were analyzed by bisulfite sequencing and shown here is the methylation status of maternal ICR. Each circle represents a CpG site with black = methylated and white = unmethylated. Percentages of methylation at all CpGs are indicated. Average CpG methylation levels are 82.0±4.3% in controls and 53.8±2.4% in upper dose (P<0.01).

To determine whether LOI observed in tissues from BPA exposed mice was linked to abnormal patterns of DNA methylation, we performed DNA methylation analysis in E9.5 embryos and placentas. We first conducted a pyrosequencing assay testing DNA methylation profiles at 7 CpG sites of the promoter region of *Snrpn*. The *Snrpn* promoter is located within an ICR that is hypermethylated on the maternal allele but hypomethylated on the paternal allele [31]. Pyrosequencing analysis of a subset of placentas (that exhibited either normal monoallelic or LOI of the *Snrpn* gene) revealed a small reduction in mean methylation levels in upper dose BPA exposed placentas (37.2±2.5%; n = 7) as compared to controls (40.2±3.3%; n = 8; P<0.05 through ANOVA; Figure 5A, 5B); however upon non-parametric analysis, this difference was not statistically significant (P>0.05 through Kruskal-Wallis). We subsequently performed bisulfite mutagenesis analysis to assay allele-specific methylation levels at 16 CpG sites at the *Snrpn* ICR (Figure 5A, 5C). In addition to examining methylation level at more CpG sites as compared to the pyrosequencing assay (16 vs. 7 sites, respectively; Materials and Methods), bisulfite mutagenesis sequencing is a more sensitive assay to detect small methylation level differences as *allele-specific* methylation, rather than *total* methylation is measured. We assayed a subset of F1 hybrid placentas from E9.5 upper dose BPA exposed and control mice (n = 3 in each group; all placentas from the upper dose group showed LOI). Consistent with previously published data [15], [31], control placentas showed the expected patterns of differential methylation, i.e., hypomethylated paternal allele (data not shown) and hypermethylated maternal allele with methylation levels at all CpG sites ranging from 77.3 to 85.7% (average of 82.0±4.3%; Figure 5C). Upper dose BPA exposed placentas also exhibited the expected hypomethylated paternal allele (data not shown) and hypermethylated maternal allele, however, methylation levels of all CpG sites of the maternal allele were lower than controls, ranging from 51.8 to 57.2% (average of 53.8±2.4%; Figure 5C; P<0.01).

In the E9.5 embryos, our pyrosequencing analysis revealed a small reduction of mean methylation levels at the 7 CpG sites in the *Snrpn* ICR (40.8±2.1% in control vs. 36.8±4.0% in upper dose BPA; n = 7 and 9, respectively; P<0.05 through ANOVA) but this difference was not significant when tested in a non-parametric method (P>0.05 through Kruskal-Wallis). Analysis on the E12.5 brain tissues revealed no difference in mean methylation between control (42.6±2.4%; n = 6) and upper dose BPA exposure (43.9±2.4; n = 9) groups despite the increased LOI rate we observed. No mean methylation difference was detected in embryos from the lower dose (37.1±9.1%; n = 14) and control groups (P = 0.16).

**Figure 6:**
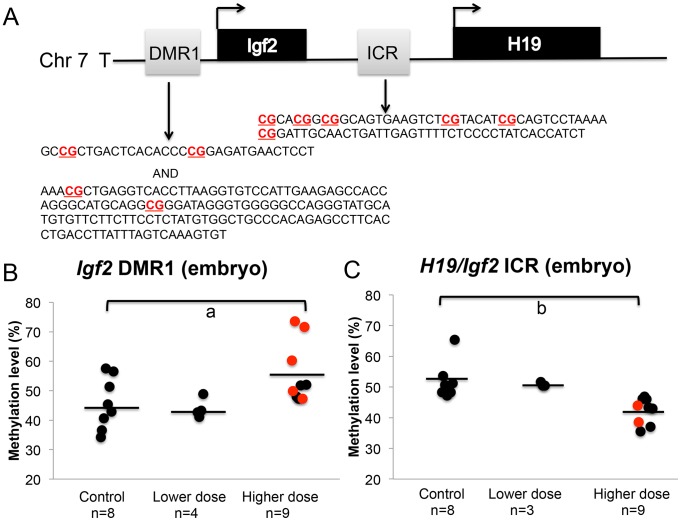
BPA exposure altered DNA methylation at the *Igf2* DMR1 and *H19/Igf2* ICR in the embryos. A. Pyrosequencing assays tested methylation of 4 and 6 CpGs (highlighted in bold) in 457 bp and 221 bp genomic regions at the *Igf2* DMR1 and *H19/Igf2* ICR, respectively. Methylation at the (B) *Igf2* DMR1 and (C) *H19/Igf2* ICR was analyzed in embryos from controls, lower dose and upper dose exposure groups. Analysis in the upper dose exposure group included embryos that showed monoallelic (black circles) or biallelic (red circles) expression of the *Igf2* gene. Sample sizes analyzed in each exposure group are indicated with a = P<0.05 and b = P<0.01. Black horizontal line indicates average methylation level in each exposure group.

We next determined whether the observed increased proportion of upper dose BPA exposed embryos exhibiting LOI of the *Igf2* gene was associated with changes in DNA methylation levels by performing pyrosequencing assays analyzing the *H19/Igf2* ICR located between the two genes and a DMR located at the promoter region of *Igf2*, the *Igf2* DMR1 (Figure 6A). Our pyrosequencing analysis for the *H19/Igf2* ICR revealed a slight but significantly reduced average methylation of the 6 CpG sites assayed in upper dose BPA exposed embryos (51.6%±5.9% in control vs. 42.3%±4.2% in upper dose BPA; n = 8 and 9, respectively; P<0.001; Figure 6C and Figure S2A). This pattern was unexpected because it was inconsistent with the *Igf2* expression profiles; biallelic *Igf2* is typically associated with gain of DNA methylation at the ICR [27]. No effect on methylation was observed in embryos from lower dose group (50.9±0.7%; n = 3). Analysis of the *Igf2* DMR1, however, was consistent with its expression profiles [32] because it showed significantly increased mean methylation at 4 CpG sites in the embryos from upper dose BPA exposed mice (45.6±8.7% in control vs. 55.7±10.3% in upper dose BPA; n = 8 and 9, respectively; P<0.05; Figure 6B and Figure S2B); no effect was observed in the lower dose group (43.9±3.3%; n = 4; Figure 6B). Neither assay revealed significant methylation changes between BPA exposed (both upper and lower doses) and control placentas (data not shown).

### Effects of BPA exposure on imprinted gene expression are more severe in the placenta compared to the embryo

**Figure 7:**
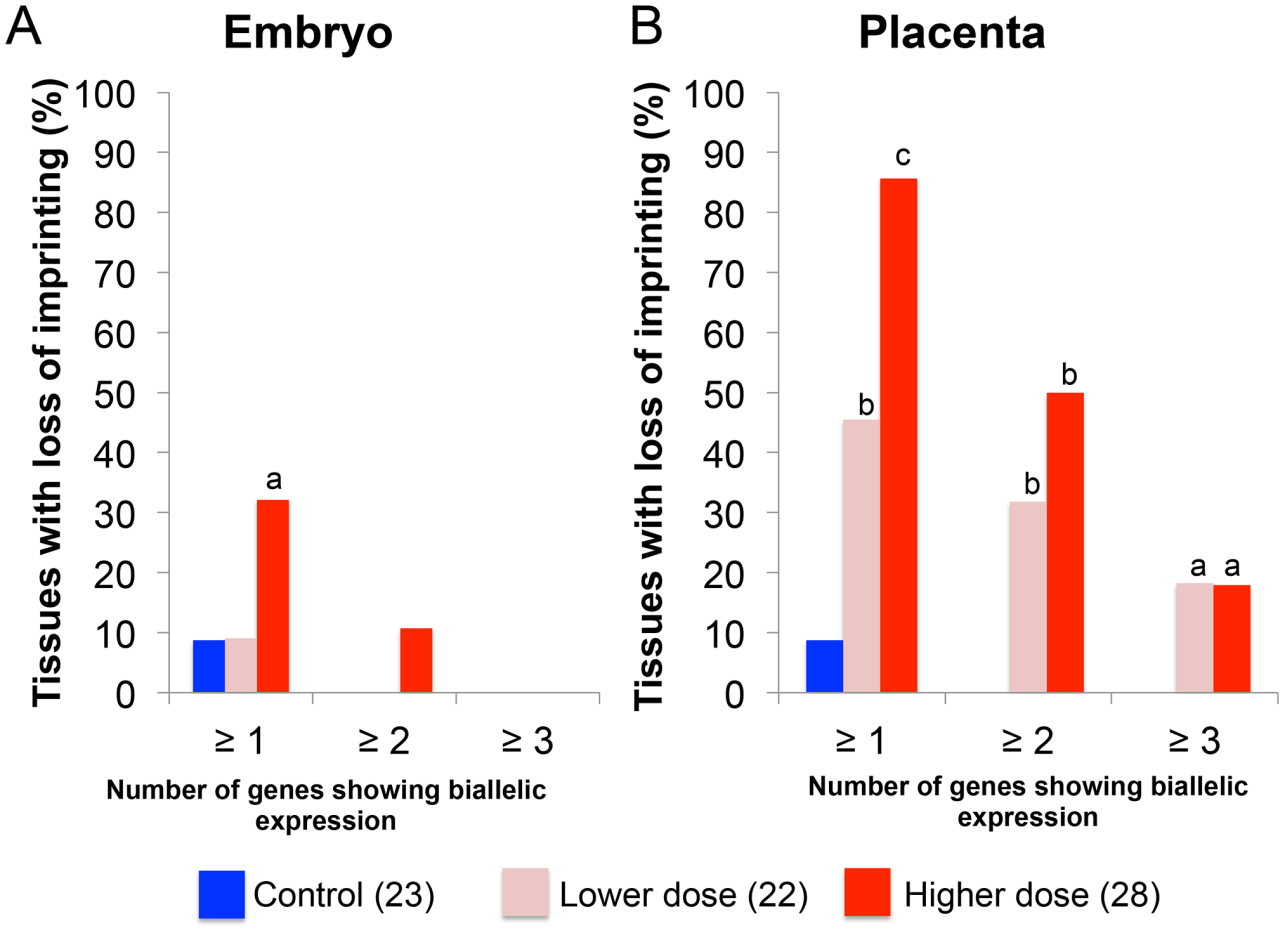
Impact of BPA exposure is shown as number of genes exhibiting imprinting perturbations. Effects of BPA exposure were more significant in the (B) placentas compared to (A) embryos. For each exposure group (control [blue], lower dose [pink] or upper dose [red]), percentage of tissues showing LOI (Y-axis) of at least 1 gene (≥1), 2 genes (≥2) or 3 genes (≥3) is indicated; a = P<0.05; b = P<0.01; c = P<0.001.

We subsequently determined the severity of impact of BPA exposure in the E9.5 embryo and placenta by comparing the number of imprinted genes exhibiting LOI in each tissue. In the embryo, 32.1% (n = 28) of upper dose BPA samples exhibited LOI of at least 1 imprinted gene compared to 8.7% in control (n = 23; P<0.05; Figure 7A). Impact of lower dose exposure did not differ significantly from controls (8.7%; n = 22; Figure 7A). The percentage of embryos showing LOI of 2 genes or more in control (0.0%) and upper dose BPA (10.7%) exposure groups was not statistically different (Figure 7A).

BPA exposure affected the E9.5 placenta more severely with 85.7% and 45.5% of samples in the upper dose (n = 28) and lower dose (n = 22) exposure groups, respectively, showing LOI of at least 1 imprinted gene compared to 8.7% in controls (n = 23; P<0.0001 and P<0.01, respectively; Figure 7B). The difference between severity of LOI in BPA exposed and control groups was still statistically significant when 2 imprinted genes or more were considered (50.0% and 31.8% of placentas from upper dose and lower dose exposure groups, respectively, compared to 0.0% in controls; P<0.01; Figure 7B) or even for 3 imprinted genes or more (17.9% and 18.2% of placentas from upper dose and lower dose exposure groups, respectively, compared to 0.0% in controls; P<0.05; Figure 7B).

### BPA exposed placentas exhibit reduced global DNA methylation

**Figure 8:**
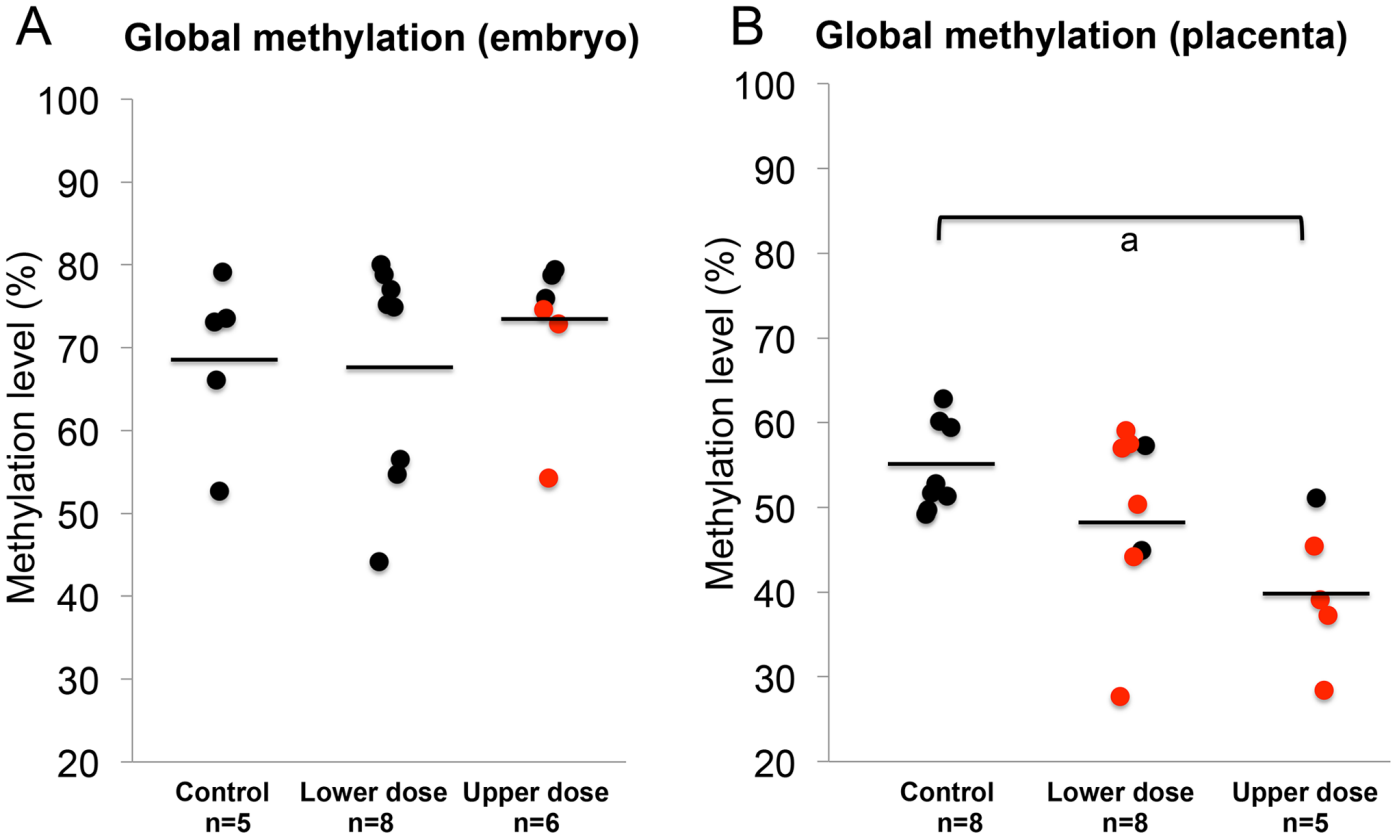
BPA exposure reduced genome-wide DNA methylation in the placenta but not the embryo. Results of LUMA studies showed genome-wide DNA methylation levels in the (A) embryos and (B) placentas from control, lower dose and upper dose BPA exposure groups. Black circles indicate tissues with normal, monoallelic expression of all imprinted genes tested; red circles are tissues that showed LOI of at least 1 imprinted gene. Black horizontal lines represent average total methylation in each exposure group; a = P<0.05.

The observed effects of BPA exposure on imprinted gene expression and DNA methylation as well as differences in the severity of BPA effects in the embryo vs. placenta prompted us to investigate effects of exposure on genome wide DNA methylation and to determine whether the embryo and placenta displayed significant differences. We performed experiments using the Luminometric Methylation Assay (LUMA) in control and BPA exposed embryos and placentas. The method estimates genome wide DNA methylation based on combined DNA cleavage by restriction endonucleases followed by a polymerase extension assay using the pyrosequencer [33]. Our data showed that upper dose BPA exposure significantly reduced the average global methylation of the placenta at E9.5 (54.6±5.3% vs. 40.3±5.9% in the control vs. upper dose BPA exposed, P<0.05; with n = 8 and 5, respectively; Figure 8B and Figure S2C). Average DNA methylation difference between lower dose and control groups was not statistically different (49.8±10.6% vs. 54.6±5.3%, respectively; n = 8 and 7; Figure 8B). No significant differences in methylation were seen in the E9.5 embryo (68.9±10.2% in control, 67.7±13.7% in lower dose and 72.7±9.3% in upper dose BPA exposure groups; n = 5, 8 and 6, respectively; Figure 8A). Similar observations were observed in the embryos and placentas at E12.5 (data not shown). We note that our global methylation data in control tissues were consistent with previous publications: Gallou-Kabani et al., (2010) reported similar average DNA methylation in E15.5 placentas using the LUMA although levels in the embryo were not reported [17]. Additionally, Popp et al., (2010) reported similar levels of average DNA methylation in E13.5 embryos and placentas as well as highly variable individual global methylation in their genome-wide bisulfite sequencing analysis [34].

### Upper dose BPA exposure results in abnormal placentation

Our data suggested that the placenta was a significant tissue target for BPA induced epigenetic perturbations as indicated by aberrant expression and methylation of imprinted genes located in the *Snrpn* and *Kcnq1* domains as well as significantly reduced genome-wide methylation. We further tested whether upper dose BPA exposure affected placenta-specific imprinted genes by studying the allelic expression patterns of the placenta-specific imprinted genes *Ascl2* and *Tssc4* located in the *Kcnq1* domain (Figure 2). Analysis of *Ascl2* revealed that a significant proportion of upper dose BPA exposed E9.5 placentas showed biallelic expression (6/24) as compared to controls (0/16; P<0.05) with expression of the normally repressed maternal allele ranging from 16.4–43.7% (Figure 3D). No significant effects were observed at the *Ascl2* gene in the lower dose group (1/16; Figure 3D). At the *Tssc4* locus, a trend towards increased rate of LOI was observed in the upper dose but was not statistically significant (Table S1).

**Figure 9:**
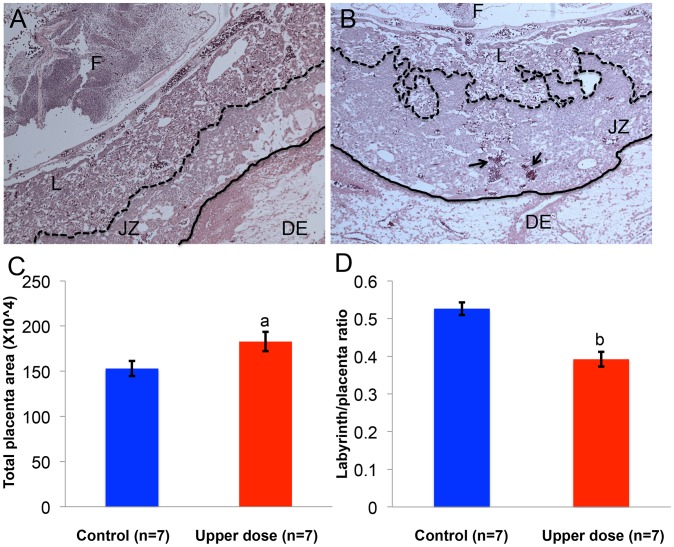
BPA exposure was associated with defective placental development. Hematoxylin and eosin-stained histological sections of representative E12.5 conceptuses from (A) control and (B) upper dose BPA exposure groups are shown. F = fetus; DE = maternal decidua; L = labyrinth zone and JZ = junctional zone. Dotted line indicates the boundary between JZ and L, continuous line the boundary between JZ and DE, and arrows the accumulation of red blood cells. Total placenta area (C) and the ratio of area occupied by the labyrinth zone and total placenta area (D) were measured in both control (blue) and upper dose BPA exposed (red) placentas; a = P<0.05; b = P<0.001.

As imprinted genes play a critical role in placental development [35], we determined whether aberrant imprinting induced by BPA exposure was associated with abnormal placental phenotypes. We performed histological and immunohistochemical examinations on placentas from the upper dose and control exposure groups. Because the major placental zones (i.e., the labyrinth and junctional zones) have just begun to develop at E9.5 [36], histology and immunohistochemistry were conducted at E12.5. In both control and upper dose exposure groups, the junctional zone and labyrinth were easily distinguishable (Figure 9A, 9B). We observed, however, that the placentas from upper dose exposure group (n = 7) had significantly larger total area (182.7±10.6 µm^2^ vs. 152.9±8.3 µm^2^; P<0.05; Figure 9C) but smaller labyrinth/placenta ratio (0.39±0.02 vs. 0.52±0.02; P<0.001; Figure 9D) compared to controls (n = 7). Additionally, BPA exposed placentas had distorted boundary between the junctional zone and labyrinth and increased accumulation of red blood cells (Figure 9B). There was no significant difference in the maternal decidua/placenta ratio between control and upper dose BPA exposed placentas (data not shown).

## Discussion

In this work, we performed an extensive characterization of the effects of environmental exposures on epigenetic regulation of developmental genes. We analyzed impact of fetal BPA exposure on imprinted gene expression and methylation in mouse embryos and placentas. As BPA exposure alters DNA methylation status [9], [22]–[24], we hypothesized that exposure perturbed genomic imprinting and disrupted embryonic and/or placental development. We analyzed E9.5 and E12.5 embryos and placentas from BPA exposed conceptuses for evidence of imprinting perturbations. Recently, Szabo and coworkers [37] investigated effects of exposure to 200 µg/kg/day of BPA on genomic imprinting but reported mild perturbations in E13.5 mouse embryonic and extraembryonic tissues. Their exposure, however, occurred between E8.5 and E12.5, during the period when germ cells are migrating and beginning to reprogram [38]. Our maternal exposure initiated 2 weeks prior to mating until pregnancy day 9.5 and 12.5, coincided with the epigenetic reprogramming of the genome during late oocyte maturation and post-fertilization development [13]. This exposure window includes the period when methylation is being established in the oocyte and post fertilization when the majority of the genome is reprogrammed [29]. Importantly, genomic imprints of ICRs are maintained during this time. Because Szabo and coworkers reported no significant effects on the *Snrpn, Kcnq1ot1* and *Ascl2* genes, the findings of LOI and/or DNA methylation changes at these loci in this current study suggest that imprinting regulation is more vulnerable to environmental exposure during the epigenetic reprogramming period that coincides with the establishment and maintenance of DNA methylation [6]–[10] as compared to during mid-gestation. Consistent with this observation, BPA exposure outside of the epigenetic reprogramming window, i.e., from E5.5–E12.5, in the current study did not lead to significant imprinting perturbations. Additionally, differences in doses, mouse strains and route of administrations between the two studies may explain differences in results. Neither study found significant effects at the *H19* and *Peg3* loci.

Our work has demonstrated that BPA exposure disrupted imprinted gene regulation, with the *Snrpn* and *Kcnq1* domains significantly affected in the placenta while the *Igf2* gene imprinting was disrupted exclusively in the embryo. Our findings support previous studies demonstrating the ability of environmental factors to impact genomic imprinting regulation in early mouse development [14]–[17], although unlike these studies [14]–[16], we did not observe significant effects at the *H19* gene. Furthermore, ART-associated environmental perturbations are applicable to patients undergoing ART procedures [14]–[16] and therefore, the potential health consequences are limited to the babies conceived through this technology; in contrast, the environmental factor investigated in the current study is present ubiquitously and the potential risks of exposure are applicable to the general population.

Importantly, our observations at the *Snrpn* domain recapitulate the molecular signatures of Angelman Syndrome (AS) in humans, of which a significant proportion of cases are caused by absence of the E3A ubiquitin ligase gene *UBE3A* product due to mutation or epigenetic aberrations at the *SNRPN* imprinting center (IC) in the brain [39], [40]. In the brain of AS mouse models carrying insertion/duplication mutation of a region analogous to human AS IC, the maternal *Snrpn* promoter had reduced DNA methylation, biallelic *Snrpn* gene expression and reduced total expression of the *Ube3a* gene [40]. Given the potentially reduced DNA methylation of the ICR at this locus in the E9.5 whole embryo, significant increased LOI rate in the E12.5 brain as well as lower expression of *Ube3a* following BPA exposure in our study, these results raise the possibility that environmental perturbations could contribute to the rare cases of AS caused by loss of methylation at the IC [40]. Although the environment has been postulated to influence incidence of imprinting diseases [41], our study is the first to suggest that exposure to a man-made compound may be implicated.

Similar to previous studies [15], [16], we found the placenta to exhibit more significant imprinting perturbations than the whole embryo (Figure 7A, 7B). One possible explanation is that the placenta is in direct contact with maternal tissue causing it to be more highly exposed to the environment. Blood measurement in humans has found more accumulation of BPA in the placenta compared to the fetus [42]. However, in the current study, the placentas and/or embryos from BPA exposed mice exhibited significant changes in DNA methylation at DMRs, including the *Snrpn* ICR and *Igf2* DMR1 (Figure 5A, 5B), suggesting that the embryo was not protected from environmental insults. Normal, monoallelic expression of *Snrpn* in BPA exposed embryos observed in our study, despite significantly reduced methylation, could reflect differences in imprinting regulation between the embryo and placenta. It is postulated that imprinting in the embryo depends largely on DNA methylation while both posttranslational histone modifications and DNA methylation play a regulatory role in the placenta [43], [44] - as DNA methylation is the primary regulator of imprinting in the embryo, it is possible that additional layers of repression are present. Alternatively, frequencies of LOI may be variable in different embryonic organs and effects of imprinting perturbations may become less significant when the whole embryo is investigated. Our allele-specific expression analysis on E12.5 brain from upper dose BPA exposure group indeed revealed increased LOI rate at the *Snrpn* locus as compared to controls, although, we found no changes in DNA methylation levels. Furthermore, it would be interesting to determine whether increased duration of exposure or combined exposure with other endocrine disruptors would produce more significant effects on imprinted gene expression at the *Snrpn* and *Kcnq1* domains in the embryo and/or organs. These paradigms would be relevant to humans as BPA is ubiquitous and exists in the presence of other environmental pollutants, and effects of these different compounds may be additive or synergistic [45].

Furthermore, we observed significantly increased LOI of the *Igf2* gene among E9.5 (Figure 3B) and E12.5 embryos (Figure 3E) exposed to 10 mg/kg bw/day of BPA and these effects were associated with increased methylation at the *Igf2* DMR1 in E9.5 embryos (Figure 5D and Figure S2B). In humans, altered methylation patterns at the *IGF2* gene are linked to environmental perturbation or intervention during early development including smoking [46], maternal starvation [47] and folic acid supplementation [48]. In humans and mice, altered *IGF2* or *Igf2* expression significantly alters growth [49]–[51]. In the current study, we did not observe significant fetal body weight differences between controls and BPA exposed mice at E9.5 and E12.5 (data not shown), although it was possible that these stages were too early to assess growth effects. Alternatively, the abnormal placental development in our BPA exposed mice may have counteracted the effect of increased *Igf2* expression.

We found that although BPA exposure significantly altered average methylation levels at various DMRs, both allelic and total expression data were not perfectly correlated with each other or with methylation changes (e.g., total expression changes may occur without LOI or hypomethylation at the *Snrpn* ICR was not always associated with LOI or vice versa). The observation, however, has been previously noted in studies of *Dnmt1* mouse mutants in which allelic expression changes in the embryos and placentas were not always associated with comparable changes in total expression [52]. Additionally, Weaver et al (2010) also observed that loss of methylation in *Dnmt1* mouse mutants did not always cause LOI [52]. Lack of perfect correlation between methylation and imprinted expression in our BPA exposure model suggests that other epigenetic mechanisms in addition to DNA methylation could be involved in mediating BPA-induced imprinting aberrations. Additionally, most imprinted genes are expressed only in a subset of cells in the embryo and placenta, making a 1 to 1 correlation in a complex tissue very difficult.

One important finding of our study is that lower dose BPA exposure was sufficient to induce significant epigenetic changes. We initially employed an exposure level of 10 mg/kg bw/day BPA as a starting point because Dolinoy et al. reported significant effects on DNA methylation in the *agouti* mice when exposed to this dose [9]. To our surprise, a 1000-fold lower dose of 10 µg/kg bw/day BPA was sufficient to alter imprinting at the *Snrpn* and *Kcnq1* domains in E9.5 placentas (Figure 3A, 3C). This lower dose corresponds to what is considered a safe human exposure level (i.e., determined as ≤50 µg/kg bw/day [20]). Although the lower dose does not result in a significantly different serum BPA level compared to controls (presumably due to the difficulty in assaying this level of BPA), the average serum BPA levels measured in pregnant mice treated with 10 mg/kg bw/day of BPA in this work was consistent with previous studies employing similar paradigm [53]. Moreover, the detected serum levels were within the range of BPA concentrations measured in human blood [20], suggesting that our exposure paradigm was biologically relevant to humans. Thus, our studies are consistent with lower dose BPA scientific literature that reports a wide variety of developmental defects related to metabolism, immune system, reproduction and brain function [21] following exposure at or below the determined “safe” human exposure level.

Our placenta studies revealed that upper dose BPA exposure was associated with abnormal development characterized by significantly larger placenta area and smaller labyrinth to placenta ratio (Figure 9A–9D) that may have arisen due to overgrowth of the junctional zone and/or reduced size of the labyrinth. Tachibana et al (2007) have previously reported effects of BPA exposure in placental development with similar findings of reduced proportion of labyrinth layer in the ICR mouse strain [54]. To our knowledge, however, the current study is the first to suggest that the placental phenotype induced by environmental exposures may be associated with epigenetic perturbations. Our findings are consistent with other mouse studies reporting similar placental phenotypes including overgrowth that are linked to defective epigenetic reprogramming including in mice produced by embryo transfer [55] or mice with aberrations in imprinting due to paternal uniparental disomy of chromosome 12 [56]. Furthermore, loss of function of the cyclin dependent kinase *Cdkn1c* gene in the mouse causes larger placentas due to overgrowth of the trophoblast cells [57]; the larger placenta area and reduced ratio of labyrinth/placenta observed in the current study may be partly explained by the significantly lower total expression of the *Cdkn1c* gene in our BPA exposed placentas. As placentomegaly is often observed in the human overgrowth disease, BWS, in which 10% of cases are attributed to loss of function or mutation in the *CDKN1C* gene [27], results of our work demonstrate that disruption of this pathway due to environmental exposures may be associated with adverse placental development. The placental phenotype observed in the current study may also be the result of perturbed non-imprinted genes that play a role in placental development as our genome wide methylation analysis indicated significantly reduced methylation levels in BPA-exposed placentas. Future studies aiming at the identification of exact mechanistic links between exposure and placental defects as well as detailed examinations at the placenta at various developmental stages would be critical.

In the mouse, maternal-fetal material exchanges including O_2_ occur in the labyrinth [36]. Because hypoxia is commonly linked to formation of new blood vessels in the placenta [58], the observation of increased accumulation of red blood cells in our BPA exposed placentas (Figure 9B) suggests that the reduced proportion of labyrinth may have affected oxygenation. This observation needs to be investigated carefully in the future as its implication is directly relevant to placental function and fetal health. Lack of O_2_ at later stages of placental development restricts vascularization of the labyrinth and is potentially associated with intrauterine growth restriction (IUGR) [59]. Although human placenta is structurally distinct from mouse, mouse labyrinth layer is functionally analogous to the chorionic villi of human placenta and certain severe cases of early onset IUGR and spontaneous abortion can be attributed to its dysfunction [36]. The placentation defect in the current study may explain the absence of weight increase in BPA exposed embryos despite increased total expression of *Igf2* as discussed above.

The mechanisms involved in BPA induced epigenetic changes are currently under investigation. The best-characterized property of BPA is its ability to act as an estrogen and increasing data have shown that some relevant developmental effects are mediated through estrogen receptors (ERs) [21]. Role of estrogen in DNA methylation-dependent epigenetic regulation has been suggested [60] and BPA exposure has recently been shown to decrease or increase expression levels of the *Dnmt3a/b* and/or methyl CpG binding proteins (*Mbd)2/4* genes [61], [62]. BPA may therefore induce hypomethylation or hypermethylation in exposed rodent models [9], [61], [62] through alterations in the genes responsible for DNA methylation maintenance using estrogen-dependent signaling pathways.

As imprinting regulation between mice and humans shares similar features, results of the current work have important implications for human health. DNA methylation defects are often observed in human imprinting disorders, for example a significant proportion of BWS cases arises from abnormal methylation at the *H19/IGF2* ICR or epigenetic defects at the ICR of *KCNQ1* [27]. The current study found that environmental exposure induced methylation changes at the DMRs of imprinted domains, suggesting that it is highly likely that the environment may play a role in the etiology of imprinting diseases.

Furthermore, our work suggests that BPA exposure can perturb epigenetic regulation of developmental genes that play significant roles in neurobehavioral development or growth. Increasing evidence has linked imprinted genes to brain function [63], [64] and disrupted imprinting causes neurodevelopmental disorders including AS and PWS. As maternal BPA levels in humans are linked to neurobehavioral development in children including behavioral and emotional functions [65]–[67], it would be critical to determine whether endocrine disruptors including BPA may influence brain and behavior functions through perturbed imprinting regulation or aberrant function of other developmental genes that are subject to epigenetic regulation. Additionally, BPA exposure is linked to alteration in body weight of the offspring in animal studies [68], [69]. These findings are consistent with epidemiological observations in humans suggesting the association between BPA exposure and preterm birth [70], or small for gestation age [71]. Our work demonstrated that BPA exposure can potentially influence growth and development by altering expression of the growth-promoting gene *Igf2* or through disruption of placental development.

In summary, our work has demonstrated the ability of exposure to the common chemical BPA to disrupt epigenetic regulation of developmentally relevant genes. We have shown that exposure to physiological relevant doses of BPA perturbs expression and methylation of imprinted genes in the mouse with the most significant effects observed in the placenta and that the effects were associated with abnormal placental development. The current study found that early embryonic development is susceptible to environmental perturbations, and we have shown for the first time that man-made compounds are potentially capable to alter epigenetic reprograming events. In humans, this sensitive developmental window coincides with the earliest stage of pregnancy at the time when it is not yet clinically recognized, thus, to reduce potential BPA exposure-induced health abnormalities, exposure management should begin even before a woman gets pregnant. Unfortunately, due to the ubiquitous nature of BPA, complete avoidance is almost impossible as most individuals in the US are exposed [20]. In the future, studies should be performed to address whether BPA-induced defects can alter placental function and whether the dysfunction leads to changes in body weight and/or other metabolic disorders. Furthermore, studies elucidating mechanisms related to BPA-induced epigenetic alterations including the utilization of the estrogen receptor knock out mice should be undertaken as results from such studies would provide critical insights into BPA related developmental abnormalities and preventive actions.

## Materials and Methods

### Mouse information

For allele-specific expression and methylation studies, we used two different mouse strains: the C57BL/6 (B6; Jackson Laboratory, Bar Harbor, ME) and the B6 (CAST7) or C7 strain [72] that contains chromosome 7 of the *Mus castaneus* (Cast) strain in a B6 background. F1 hybrid progeny, which were assayed for allelic expression and methylation, were derived from reciprocal matings between B6 and C7 mice. Polymorphisms between the two mouse strains were used to distinguish the parental origin of expression or methylation of genes of interest. All animal work was conducted with the approval of the Institutional Animal Care and Use Committee.

Virgin females, 6–10 weeks old, were assigned to one of the following diets: a) modified AIN-93G diet (diet TD.95092 with 7% corn oil substituted for 7% soybean oil; Harlan Teklad) as “control”; b) modified AIN-93G diet supplemented with 50 mg/kg diet of BPA (diet TD.06156; Harlan Teklad) as “upper dose”; or c) modified AIN-93G diet supplemented with 50 µg/kg diet of BPA (diet TD.110337; Harlan Teklad) as “lower dose”. Teklad Diets (Harlan Laboratories Inc, Madison, WI) provided all ingredients except BPA (Sigma Aldrich, St. Louis, MO). Drinking water was provided in polypropylene bottles. The estimated daily treatment of BPA in our study was 10 mg/kg body weight (bw) in the “upper dose” and 10 µg/kg bw in the “lower dose” groups. Diet was provided 2 weeks prior to mating and during pregnancy until embryonic day (E) 9.5 and E12.5. At E9.5 and E12.5, pregnant mice were euthanized and tissues from the whole embryo and placenta isolated. Prior to placental tissue isolation, the maternal decidua was carefully removed. For tissue-specific analysis, different embryonic organs including the heart, liver, kidney and brain were isolated from E12.5 mice. Upon isolation, tissues were halved (each half for expression and methylation studies) and frozen at −80°C.

### Expression studies

Total RNA was extracted using Trizol according to the manufacturer's instructions and quantified using the Nano Drop spectrophotometer. cDNA was prepared by using Superscript III reverse transcriptase and random hexamers. To test that RNA was free of genomic DNA contamination, a minus reverse transcription control was included.

#### Allele-specific expression

Allele-specific *Snrpn* and *H19* expression studies were conducted on E9.5 and E12.5 embryonic and placental cDNA using the Light Cycler real time PCR system as described previously [14], [52]. For analysis of *Igf2*, *Kcnq1ot1*, *Cdkn1c*, *Ascl2* and *Peg3*, we conducted RT-PCR followed by restriction enzymes using the conditions described previously [14],[52]. Samples that showed ≥10% of total expression derived from the repressed allele were considered to be biallelic or to exhibit loss of imprinting (LOI).

#### Total expression

Quantification of mRNA levels was determined in E9.5 embryos and placentas using the Light Cycler Real Time PCR System (Roche); for analysis of the *Ube3a* gene, E12.5 embryonic brains were also included. The PCR conditions and primers information were described previously [14], [52]. Total expression of imprinted genes was calculated relative to the geometric mean of the reference genes *Arpp0* (acidic phosphoprotein P0 subunit*)* and *Gapdh* (glyceraldehyde-3-phosphate dehydrogenase). Samples were analyzed at least in duplicates using the Light Cycler 4.0 software as described [52].

### Methylation studies

DNA was extracted using the standard phenol chloroform method and amount measured using the Nano Drop spectrophotometer. One microgram of DNA was bisulfite treated using the EpiTect Bisulfite Kit (Qiagen) following manufacturer's protocol.

#### Pyrosequencing assays

Pyrosequencing was performed to analyze the methylation profiles at the *Snrpn* ICR (chr7: 67,149,897–67,150,141; NCBI37/MM9), *H19/Igf2* ICR (chr7: 149,767,599–149,767,819; NCBI37/MM9) and *Igf2* DMR1 (chr7: 149,851,180–149,851,655; NCBI37/MM9). 50 ng of bisulfite treated DNA was used for PCR. For *Snrpn* ICR, the primer sequences were as follows: forward primer 5′- GGTAGTTGTTTTTTGGTAGGATAT-3′, biotinylated reverse primer 5′- ACTAAAATCCACAAACCCAACTAACCT-3′. For *H19/Igf2* ICR, the primer sequences were as follows: forward primer 5′- GGGTAGGATATATGTATTTTTTAGGTTG- 3′ and biotinylated reverse primer 5′- CTCATAAAACCCATAACTATAAAATCAT- 3′. For *Igf2* DMR1, the primer sequences were as follows: forward primer 5′- TGAGGTTAGATTAGGTTGTAAGTT-3′ and biotinylated reverse primer 5′- CTTCCCTACCCCTTAAACC -3′. The PyroMark PCR kit (Qiagen) was used in a 25 µL reaction according to the manufacturer's protocol. PCR conditions were: 95°C for 15 minutes followed by 45 cycles of 95°C for 15 seconds, 55°C (or 58°C for *Igf2* DMR1) for 30 seconds and 72°C for 15 seconds. 10 µL of the biotinylated PCR product was used for each sequencing assay with the *Snrpn* sequencing primer 5′- GTGTAGTTATTGTTTGGGA- 3′, or *H19/Igf2* ICR sequencing primer 5′- TGTAAAGATTAGGGTTGT- 3′, or *Igf2* DMR1 sequencing primers 5′-GGATTTTGTTAGGTAGGA-3′ and 5′-TTTTAGAGGTTTTTGGAGAA-3′. Pyrosequencing was done using the PyroMark Q96MD (Qiagen) system following the manufacturer's protocol and the PyroMark Gold 96 reagents kit (Qiagen). Methylation was analyzed using Qiagen's Pyro Q- CpG software. 7 CpGs were analyzed for *Snrpn* ICR, 6 CpGs for *Igf2/H19* ICR, and 4 CpGs for *Igf2* DMR1. The Luminometric Methylation Assay (LUMA) was performed and analyzed as described previously [33] using 500 ng of genomic DNA.

#### Bisulfite sequencing

Bisulfite treated DNA was subject to nested PCR amplification, subcloning and sequencing of the *Snrpn* promoter-exon 1 region (2073–2601 bp, AF081460) as previously described in [72], [73]. At least 2 independent PCRs were performed on each sample. *Snrpn* parental alleles were distinguished by single nucleotide polymorphisms as previously reported [31], [72], [74].

### BPA serum measurement

Pregnant females were sacrificed on E9.5 and blood was collected through cardiac puncture, incubated at room temperature for 15 min and centrifuged at 3000 rpm. Serum was collected and stored at −80°C until analysis.

#### Materials

^13^C_12_-BPA (99% isotopic purity) used as internal standard for LC-MS analysis was purchased from Cambridge Isotope Labs (Andover, MA). 2,3,4,5,6-pentafluorobenzyl bromide (PFB-Br), sodium hydroxide and solvents were purchased from Sigma-Aldrich (St. Louis, MO). HPLC-grade hexanes, isopropanol and ethanol were obtained from Fisher Scientific Co. (Fair Lawn, NJ). ACS-grade ethanol was obtained from Pharmco (Brookfield, CT). Gases were supplied by BOC Gases (Lebanon, NJ). Chiralpak AD-H column was obtained from Chiral Technologies (West Chester, PA).

#### LC/MS conditions for BPA–PFB quantification

BPA was quantified based on a previously described procedure [75]. Normal-phase chiral chromatography for LC/MS experiments was performed using a Waters Alliance 2690 HPLC system (Waters Corp., Milford, MA). Gradient elution was performed in the linear mode. A Chiralpak AD-H column (250×4.6 mm i.d., 5 µm; Daicel Chemical Industries, Ltd., Tokyo, Japan) was employed with a flow rate of 1 mL/min. Solvent A was hexanes and solvent B was 2-propanol/methanol (1/1, v/v). The linear gradient was as follows: 2% B at 0 min, 2% B at 2 min, 50% B at 14 min, 50% B at 16 min, 2% B at 17 min, and 2% B at 25 min. The separation was performed at 30°C.

The following SRM transitions were monitored: BPA-PFB, *m/z* 227 → 113 (collision energy 28 eV), *m/z* 227 → 212 (collision energy 25 eV), [^13^C_12_]-BPA-PFB, *m/z* 239 → 139 (collision energy 28 eV) and ^13^[C_12_]-BPA-PFB, *m/z* 239 → 224 (collision energy 25 eV).

#### BPA extraction from serum samples

Serum collected from pregnant females was measured with a glass syringe and transferred to a 10 mL glass test tube containing 1 mL of Optima water spiked with 2 ng of ^13^[C_12_]-BPA (20 µL×100 pg/µL sol in acetonitrile). For blank controls, the same procedures were performed with 40 µL of water. After equilibrating for 10 min at room temperature, the samples were extracted with ethyl ether (3 mL). The organic layer was then evaporated to dryness under nitrogen, and the BPA in the residue was converted to PFB derivatives as described in [75]. The alcohol moiety was derivatized as PFB ether under strong basic conditions (sodium hydroxide). The mass spectrum obtained by CID and MS/MS analysis of [M-PFB]^−^ was identical with the underivatized BPA (spectrum not shown). However, the retention time was different on the LC system, with the PFB derivative eluting earlier (7.7 min vs. 12.7 min) than the underivatized BPA. The detection limit for the BPA-PFB was 3 pg on column.

#### Data analysis

The final concentration of BPA in serum was determined by measuring the area ratio of BPA over ^13^C_12_-BPA peaks, calculated based on the amount of ^13^C_12_-BPA added and adjusted based on the volume. All data analysis was performed using Xcalibur software, version 2.0 SR2 (Thermo Electron Corporation) from raw mass spectral data.

### Placenta analysis

At E12.5, pregnant mice were sacrificed by CO_2_ asphyxiation, and the gravid uterine horns removed from the abdominal cavity via laparotomy incision. The two most proximal gestations from each horn were excised en bloc between adjacent implantation sites with the surrounding myometrium intact and immersed in 10% phosphate buffered formalin for histological examination. The remaining conceptuses were carefully dissected from the surrounding myometrium. The amniotic membranes ruptured and separated from the placenta. Fetuses and placentas were snap frozen in liquid nitrogen and stored at −80°C for future studies.

#### Placental histology and morphometric analysis

Tissues were fixed overnight at 4°C, dehydrated, and then bivalved in sagittal section through the mid placental plane. Care was taken to place all tissues in similar orientation prior to embedding. Tissues were embedded in a paraffin block using standard protocols. Serial tissue sections, 4 µm thick, were cut through the mid placenta in sagittal section and stained with hematoxylin and eosin. Images were collected on a Leica DMRBE upright widefield microscope with a 2.5x, 5x, and 10x objective, a 5.0 MegaPixel color CCD camera, and iVision acquisition software (BioVision Technologies). Micrographs were analyzed with ImageJ v1.4.5 (NIH). All measurements were made on multiple sections through the mid sagittal plane of the placenta. The total cross-sectional area of the placenta was outlined from its base at the chorioallantois to the giant cell layer, identified morphologically. The total cross-sectional area of the labyrinth was outlined based on its characteristic morphological features. Ratios of the area of the labyrinth to the placenta as well as maternal decidua to the placenta were then calculated.

### Statistical analysis

Goodness-of-fit analyses were used to assess differences in the proportion of tissues exhibiting loss of imprinting or biallelic expression of imprinted genes. Fisher's exact test of independence was used to evaluate litter-specific effects. To evaluate the statistical differences in serum BPA levels and areas of the major placental zones, we performed standard *t*-test analyses. To test for differences in total imprinted gene expression and levels of methylation, we used the PRISM software to perform one-way analysis of variance (ANOVA) and Tukey's posttest. When indicated, expression and methylation data had been additionally analyzed using the non-parametric Kruskal-Wallis test and the results were presented as supplementary materials (Figures S1, S2).

## Supporting Information

Figure S1 Total expression of imprinted genes analyzed by a non-parametric test. Kruskal-Wallis statistical test was conducted to measure differences among exposure groups on expression of the (A) *Snrpn*, (C) *Kcnq1ot1*, (D) *Cdkn1c* and (E) *Ube3a* genes in the E9.5 placenta, (B) *Igf2* gene in the E9.5 embryo and (F) *Ube3a* gene in the E12.5 brain. Y axis = relative expression as compared to reference genes; X axis = measurement values of relative expression ranked from lowest (left) to highest (right). P values shown represent all exposure groups analyzed. P values between control and upper dose are as followed: (A) 0.006, (B) 0.05, (C) 0.04, (D) 0.002, and (E) 0.003. P values between control and lower dose are 0.003 in (C) and 0.001 in (D).
(TIF)

Figure S2 Imprinted genes-specific and genome-wide methylation levels analyzed by a non-parametric test. Results from the Kruskal-Wallis test are presented for methylation levels of the (A) *H19/Igf2* ICR and (B) *Igf2* DMR1 in the E9.5 embryos and (C) genome-wide methylation levels as measured by LUMA in the E9.5 placentas. Y = percentage of methylation level; X axis = measurement values of methylation ranked from lowest (left) to highest (right). P values shown represent all exposure groups analyzed. P values between control and upper dose are as followed: (A) 0.0006, (B) 0.04 and (C) 0.02.
(TIF)

Table S1 Percentage of total expression of imprinted genes derived from the repressed allele in each exposure group in the E9.5 embryo and placenta.
(XLS)

Table S2 Percentage of total expression of imprinted genes derived from the repressed allele in each exposure group in the E12.5 embryo and placenta.
(XLS)
