# Supplementary figures and images for: Bisphenol A Exposure Disrupts Genomic Imprinting in the Mouse
Source: PLoS Genet. 2013 Apr 4;9(4):e1003401. doi: 10.1371/journal.pgen.1003401 (PMC3616904; doi:10.1371/journal.pgen.1003401)

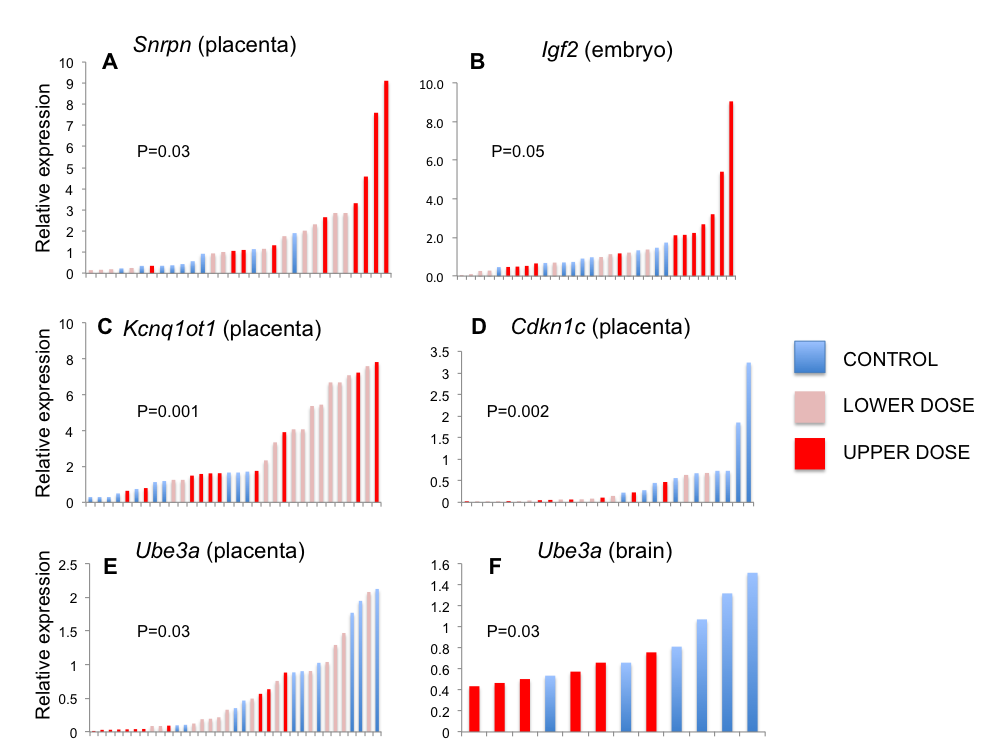

Supplement: Figure S1 — Total expression of imprinted genes analyzed by a non-parametric test. Kruskal-Wallis statistical test was conducted to measure differences among exposure groups on expression of the (A) Snrpn, (C) Kcnq1ot1, (D) Cdkn1c and (E) Ube3a genes in the E9.5 placenta, (B) Igf2 gene in the E9.5 embryo and (F) Ube3a gene in the E12.5 brain. Y axis = relative expression as compared to reference genes; X axis = measurement values of relative expression ranked from lowest (left) to highest (right). P values shown represent all exposure groups analyzed. P values between control and upper dose are as followed: (A) 0.006, (B) 0.05, (C) 0.04, (D) 0.002, and (E) 0.003. P values between control and lower dose are 0.003 in (C) and 0.001 in (D). (TIF) [file pgen.1003401.s001.tif]

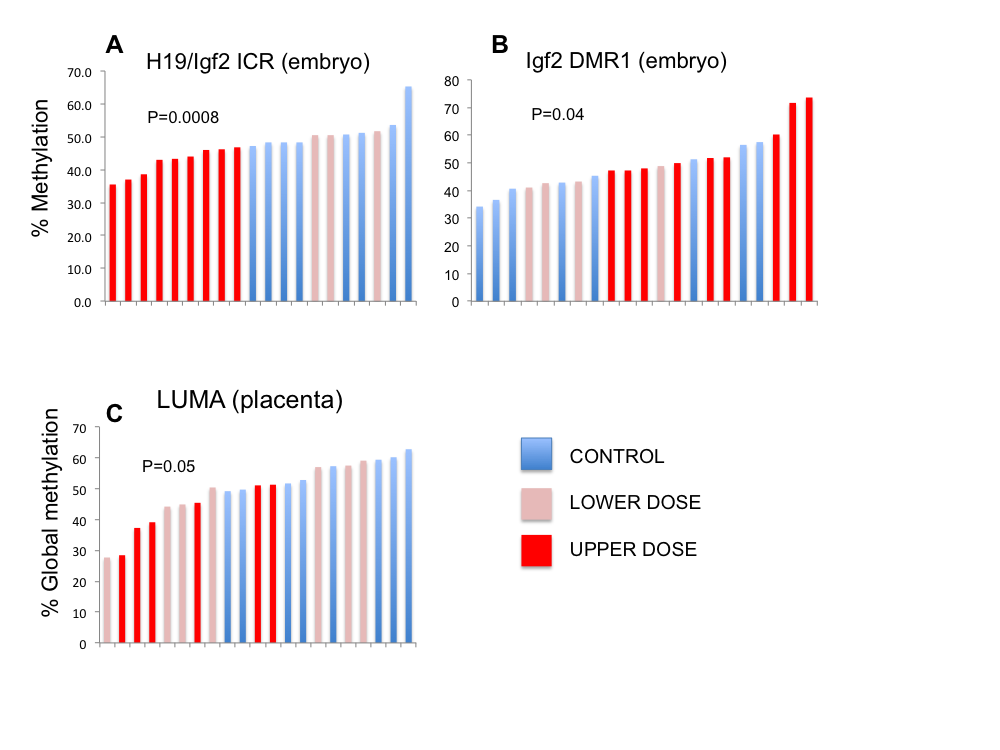

Supplement: Figure S2 — Imprinted genes-specific and genome-wide methylation levels analyzed by a non-parametric test. Results from the Kruskal-Wallis test are presented for methylation levels of the (A) H19/Igf2 ICR and (B) Igf2 DMR1 in the E9.5 embryos and (C) genome-wide methylation levels as measured by LUMA in the E9.5 placentas. Y = percentage of methylation level; X axis = measurement values of methylation ranked from lowest (left) to highest (right). P values shown represent all exposure groups analyzed. P values between control and upper dose are as followed: (A) 0.0006, (B) 0.04 and (C) 0.02. (TIF) [file pgen.1003401.s002.tif]
